# Gender Representation in Authorship of Academic Dermatology Publications During the COVID-19 Pandemic: Cross-Sectional Study

**DOI:** 10.2196/50396

**Published:** 2024-10-16

**Authors:** Mindy D Szeto, Melissa R Laughter, Mayra B C Maymone, Payal M Patel, Torunn E Sivesind, Colby L Presley, Steven M Lada, Kayd J Pulsipher, Henriette De La Garza, Robert P Dellavalle

**Affiliations:** 1 Department of Dermatology, University of Minnesota Medical School, 1-411 Phillips-Wangensteen Building, 516 Delaware St SE, MMC 98, Minneapolis, MN, 55455, United States; 2 Department of Dermatology, New York University Grossman School of Medicine, New York, NY, United States; 3 Department of Dermatology, Warren Alpert Medical School at Brown University, Providence, RI, United States; 4 Department of Dermatology, Cutaneous Biology Research Center, Massachusetts General Hospital, Harvard Medical School, Boston, MA, United States; 5 Department of Dermatology, University of Colorado Anschutz Medical Campus, Aurora, CO, United States; 6 Department of Dermatology, Lehigh Valley Network, Allentown, PA, United States; 7 Department of Emergency Medicine, Southwest Healthcare Medical Education Consortium, Temecula, CA, United States; 8 Department of Dermatology, Campbell University at Sampson Regional Medical Center, Clinton, NC, United States; 9 Department of Internal Medicine, MetroWest Medical Center, Framingham, MA, United States

**Keywords:** women, gender, representation, authorship, academic, leadership, diversity, equity, inclusion, dermatology, journals, publications, COVID-19, pandemic, bibliometric

## Abstract

Analyses of women dermatology literature authorship from 2018 to 2022 reveal a slight increase in total female authors, female first authors, and female senior authors with no substantial immediate impact of COVID-19 on current trends, encouraging future examination of long-term effects and ongoing promotion of systemic initiatives to support gender equity.

## Introduction

Early examination of the COVID-19 pandemic’s impact on academic productivity affirmed that female academics were disproportionately affected relative to their male counterparts, likely due to differential burdens including childcare and domestic demands [1]. These differences were particularly pronounced in academic medicine, where publications by women authors decreased substantially [2]. This trend has yet to be examined in-depth within dermatology. We, therefore, surveyed the representation of total female authors, female first authors (FFAs), and female senior or last authors (FSAs; a potential indicator of academics more advanced in their careers [3]) in the recent dermatologic literature.

## Methods

Dermatology articles, letters, reviews, and editorials published in 2018 through 2022 were searched on June 21, 2023, in Clarivate’s Web of Science and filtered for the top five dermatology journals by the 2022 h-index [4]. The *Journal of the American Academy of Dermatology*, the *Journal of Investigative Dermatology*, and *JAMA Dermatology* were included in the analysis, while the *British Journal of Dermatology* was excluded due to the unavailability of authors’ full first names in database citations. Binary (women vs men) gender estimation by authors’ first names was performed by genderize.io, a popular probabilistic gender inference service built on a large international database of gender-name associations collected from various web sources. The percentages of total female authors, FFAs, and FSAs were calculated for each year to allow comparisons before and during the pandemic-affected time frame.

## Results

The total proportion of female authorship increased from 41.7% (3896/9344) in 2018 to 45.2% (5214/11,536) in 2022 (*r*=0.92; Table 1). The percentages of FFAs fluctuated but slightly increased from 45.1% (771/1710) to 47% (924/1964; *r*=0.61), while the percentage of FSAs trended weakly upward over time, with a peak in 2021 at 36.4% (920/2526) and falling to 34.5% (678/1964) in 2022 (*r*=0.76). Notably, the number of total publications and authors increased each year within the four journals analyzed until peaking in 2021 (n=2526 publications totaling n=14,247 authors).

**Table 1.** Frequencies and percentages of first, senior, and total authors by gender from 2018 to 2022 in the top h-index dermatology journals.

Year		Total publications, n	Female authors, n (%)		Male authors, n (%)		Unknown authors, n (%)	
**First authors**								
	2018	1710	771 (45.1)		905 (52.9)		34 (2.0)	
	2019	1753	828 (47.2)		882 (50.3)		43 (2.5)	
	2020	2169	991 (45.7)		1118 (51.5)		60 (2.8)	
	2021	2526	1187 (47.0)		1286 (50.9)		53 (2.1)	
	2022	1964	924 (47.0)		996 (50.7)		44 (2.2)	
**Senior authors**								
	2018	1710	542 (31.7)		1121 (65.6)		47 (2.7)	
	2019	1753	576 (32.9)		1117 (63.7)		60 (3.4)	
	2020	2169	771 (35.5)		1339 (61.7)		59 (2.7)	
	2021	2526	920 (36.4)		1531 (60.6)		75 (3.0)	
	2022	1964	678 (34.5)		1219 (62.1)		67 (3.4)	
**Total authors**								
	2018	9344	3896 (41.7)		5222 (55.9)		226 (2.4)	
	2019	10,424	4321 (41.5)		5811 (55.7)		292 (2.8)	
	2020	12,341	5470 (44.3)		6592 (53.4)		279 (2.3)	
	2021	14,247	6399 (44.9)		7485 (52.5)		363 (2.5)	
	2022	11,536	5214 (45.2)		6025 (52.2)		297 (2.6)	

## Discussion

Many possible explanations exist for this trend, which differs from observations in other fields [5]. The proportion of women in dermatology who are board-certified has grown substantially, from 24% in 1992 to 53% in 2017 [6], a growth rate possibly exceeding any negative impact of COVID-19. Decreased patient capacity at dermatology clinics, suspension of elective procedures, and a prominent shift to telemedicine may be providing more time for research. However, our study was limited to certain article types from four journals, which may be influential but not fully representative of dermatology publishing. Additionally, high-throughput inference of binary gender using genderize.io’s predictive database was used due to the thousands of author names queried, but we recognize that this is a limited approach with lower accuracy for many gender-neutral names and cultural or regional differences in naming. Furthermore, given the delayed nature of the publication process and indexing, some included works may have been completed before the pandemic, compelling the need for subsequent assessment of future trajectories.

It is promising that FFA data currently suggests proportional contributions from female lead authors, but FSA percentages are still far from gender parity, corroborating patterns of female underrepresentation in senior faculty positions and ongoing inequities in research funding and academic promotion. While COVID-19 does not appear to have immediately impacted female author contributions in dermatology beyond current trends, increased analysis and discussion will be necessary to assess the long-term effects of the pandemic, determine implications surrounding author position, and strengthen support for female academic dermatologists throughout a highly varied and interdisciplinary field. Recent investigations have revealed that only 4 of the top 50 individual most cited dermatology authors by h-index in 2020 were women, though increases in overall percentages of top women authors were also observed in prior decades [7]. Given these findings, detailed characterization of the higher representation of women in dermatology and broader trends compared to other specialties could therefore identify factors contributing to the prevention or evolution of these gender disparities over time. As initial steps, increasing the number of women in academic leadership, prioritizing family-friendly work hour flexibility, and preventing burnout have been recommended as possible strategies to retain women dermatologists [8]. The need for women in leadership has been recognized by journals such as *JMIR Dermatology*, which has emphasized inviting women dermatologists to its editorial board to achieve gender parity [9]. Senior editors have considerable influence over journals and editorial procedures, and could help ensure diversity, equity, and inclusion in the publication process [10]. We hope that dermatology could therefore serve as a role model and set a precedent in demonstrating how proactive and intentional initiatives could address persistent systemic challenges in reaching gender equity.

## Abbreviations

FFA: female first author
FSA: female senior or last author
